# Variation in metabolic pattern regulation under hypoxic conditions: a comparative study of rodents distributed at different altitudes

**DOI:** 10.1186/s12983-025-00582-2

**Published:** 2025-10-01

**Authors:** Mengyang Li, Xiujuan Li, Yinan Zheng, Zhenlong Wang, Luye Shi

**Affiliations:** 1 School of Life Sciences, Zhengzhou University, No. 100 Kexue Road, High-Tech Development Zone of States, Zhengzhou, 450001 People’s Republic of China; 2 Institute of Strategic Planning, Chinese Academy of Environmental Planning, 15 Shixing Street, Shijingshan District, Beijing, 100041 People’s Republic of China; 3 State Key Laboratory of Genetic Resources and Evolution, Kunming Institute of Zoology, Chinese Academy of Sciences, 21 Qingsong Road, Kunming, 650201 People’s Republic of China

**Keywords:** Hypoxia, *Lasiopodomys brandtii*, Metabolic adaptability, *Neodon fuscus*, Skeletal muscle

## Abstract

Mammals dwelling at different altitudes exhibit distinct molecular mechanisms to adapt to low-oxygen environments owing to habitat-specific oxygen levels. Notably, these adaptations include energy metabolism patterns, which fundamentally sustain vital physiological functions. Skeletal muscle, a pivotal contributor to systemic energy metabolism, facilitates vertebrate body movement through the contraction and relaxation of muscle fibers and is highly dependent on mitochondrial substrate oxidation for energy production. This study focused on three rodent species inhabiting different altitudes: the Qinghai vole (*Neodon fuscus*), Brandt’s vole (*Lasiopodomys brandtii*), and Kunming mouse (*Mus musculus*). Using transcriptomics and quasi-targeted metabolomics, we systematically analyzed the differences in skeletal muscle metabolic regulation among the three rodent species before and after exposure to hypoxia, thereby revealing the underlying molecular mechanisms. In summary, *N. fuscus*, native to high-altitude environments, tended to sustain energy supplies through regulating fatty acid oxidation under low-oxygen conditions. Conversely, *L. brandtii* and *M. musculus*, acclimatized to middle- and low-altitude habitats, relied on aerobic oxidation and anaerobic glycolysis of glucose, respectively, for energy maintenance under hypoxic conditions. In addition to their differential metabolic preferences under hypoxic conditions, these three rodent species showed species-specific responses related to oxygen utilization, antioxidant defense mechanisms, and anti-inflammatory processes. This study provides insights into the metabolic response patterns of mammalian skeletal muscle under hypoxic conditions, thereby establishing a basis for future investigations on transcriptional–metabolic associations.

**Graphical abstract:**

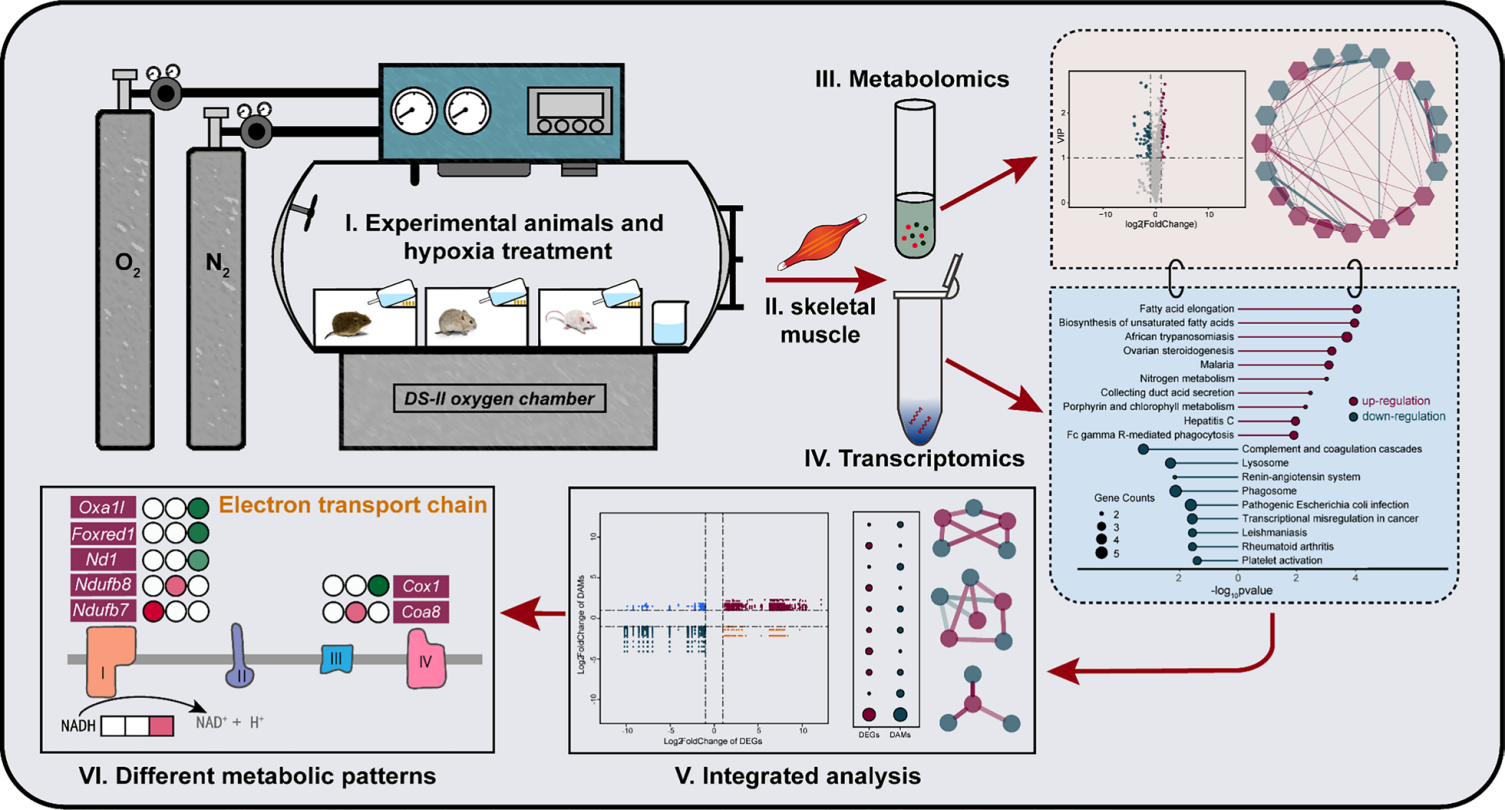

**Supplementary Information:**

The online version contains supplementary material available at 10.1186/s12983-025-00582-2.

## Introduction

Organisms have evolved complex physiological mechanisms to adapt to diverse natural environments. Among these adaptations, metabolic regulation plays a pivotal role in ensuring survival and reproduction [1–6]. Metabolic regulation involves the uptake, transformation, and expenditure of energy [7]. In general, when confronted with environmental stresses, such as hypoxia, drought, and extreme temperatures, organisms can adjust their basal metabolic rate, respiratory rate, and energy consumption as well as metabolic processing of glucose and lipids to sustain their activities [8–13].

Skeletal muscle, a striated muscle essential for maintaining physical activity and a major contributor to systemic energy metabolism [14, 15], constitutes approximately 21.6–61.4% of mammalian body mass [16, 17]. Comprising internal blood vessels, nerves, connective tissue, and multinuclear muscle fibers [18], skeletal muscle, in contrast to organs with vigorous metabolism, such as the liver [19], not only utilizes muscle glycogen to fuel its function [20], but also serves as a reservoir of amino acids for other tissues, thereby influencing the energy and protein metabolism of the entire body [21].

Given its high energy demand, skeletal muscle is highly dependent on oxygen supply to maintain energy production and redox homeostasis, and a hypoxic environment can negatively affect its normal function [22, 23]. For instance, long-term exposure to hypoxia induces progressive reductions in skeletal muscle fiber cross-sectional area as well as muscle atrophy in rats [24]. In Himalayan mountaineers, muscle cell mitochondria partially degrade into lipofuscin, resulting in the reduction of muscle oxidation capacity [25]. Environmental hypoxia also disrupts the usual aerobic metabolic process, inhibiting fatty acid oxidation while enhancing glycolysis. This phenomenon leads to significant suppression of key regulatory factors, such as peroxisome proliferator–activated receptor α (PPARα) and PPARγ coactivator-1α, for fatty acid oxidation and increase in the activity of glycolytic pathway enzymes, including hexokinase and lactate dehydrogenase [24, 26]. Consequently, this metabolic transition reduces the oxidative capacity of skeletal muscle, leading to lactic acid accumulation and diminished endurance [25].

In general, mammals residing in high-altitude regions, characterized by low-oxygen environments, exhibit superior adaptation and hypoxia tolerance in their skeletal muscles than those residing in low-altitude regions. This adaptation includes higher capillary density, mitochondrial content, myoglobin levels, and the proportion of oxidative muscle fibers [27–31]. These physiological traits are anticipated to influence metabolic performance under hypoxic conditions [32, 33]. However, the precise mechanisms through which plateau mammals and other hypoxia-exposed mammals adjust their metabolic patterns, particularly in terms of glucose and lipid metabolism, warrant further investigation [34, 35].

Li et al. [23] previously reported differences in gene expression response patterns in the skeletal muscle tissues of Qinghai voles (*Neodon fuscus*; habitat altitude: 3700–4800 m), Brandt’s voles (*Lasiopodomys brandtii*; habitat altitude: < 2000 m), and Kunming mouse (*Mus musculus*) under hypoxic conditions. Core genes associated with glucose and fatty acid oxidation, such as *Acot4*, *Acs16*, *Gpat4*, and *Rpe*, displayed distinct expression patterns across species. However, owing to the limitations of interpreting metabolic pathways solely based on gene expression, the responses of skeletal muscle in mammals with varying oxygen availability in their habitats, such as *N. fuscus*, *L. brandtii*, and *M. musculus*, to hypoxia in terms of metabolic patterns remain unclear. In contrast to transcriptomics, metabolomics, specifically quasitargeted metabolomics, offers enhanced capabilities for the qualitative and quantitative analyses of low-abundance metabolites as well as for the investigation of metabolic patterns [36, 37].

In this study, we used quasi-targeted metabolomics to comprehensively assess metabolic pattern variations in the skeletal muscles of three rodent species, i.e., *N. fuscus*, *L. brandtii*, and *M. musculus*, under hypoxic conditions. In addition, we integrated these metabolomic findings with previous transcriptomic data to offer a more comprehensive understanding of skeletal muscle metabolic responses to hypoxia in mammals inhabiting oxygen-diverse environments. Our findings provide insights into the metabolic adaptations of mammalian skeletal muscles under hypoxic conditions and establish a basis for future studies on transcriptional–metabolic associations.

## Materials and methods

### Experimental animals

*N. fuscus*, *L. brandtii*, and *M. musculus* were collected from Guoluo Tibetan Autonomous Prefecture, Qinghai Province, China (N 34°8′, E 100°11′), the Chinese Academy of Agricultural Science (Beijing, China), and the Henan Experimental Animal Center (Zhengzhou, China), respectively. All animals were individually housed in polycarbonate cages (290 mm × 178 mm × 160 mm) in a controlled laboratory environment (temperature: 20–24 °C; light cycle: 12:12 h light:dark). Daily maintenance procedures included breeding cage cleaning and replenishment of water and commercial feed (mixed rat/rabbit feed, Henan Experimental Animal Center, Zhengzhou, China) to ensure animal health. The nutritional composition per 100 g feed was 17.1% protein, 13.8% fat, and 35.3% carbohydrate.

### Sample collection and metabolite extraction

Following an eight-week acclimation period, six healthy adult male individuals with similar weights from each species were randomly assigned to either the hypoxia group (10% O_2_ for 48 h) or the normoxia group (20.9% O_2_ for 48 h). The treatment protocol aligned with that of a previous study [23], DS-II oxygen chamber (Weifang Huaxin boiler oxygen chamber Manufacturing Co., Ltd) was used to simulate normobaric hypoxia environment by controlling the inflow of O_2_ and N_2_ under sea level pressure, and all animals survived the hypoxia or normoxia treatment. After hypoxia or normoxia treatment was complete, all experimental animals were deeply anesthetized with intraperitoneal injection of pentobarbital sodium (30 mg/kg). Subsequently, the skeletal muscle tissues form left hindlimb were immediately isolated, the residual blood on the surface of the tissue was cleaned with sterile normal saline, and the surface liquid was sucked out with sterile filter paper. After quick freezing in liquid nitrogen, samples were stored in a –80 °C refrigerator. During metabolite extraction, skeletal muscle tissues (100 mg) from three animals per group were homogenized in liquid nitrogen. Prechilled 500 μL 80% methanol and a 0.1% formic acid aqueous solution were added to the homogenate, followed by thorough vortexing. The mixed samples were then incubated on ice for 5 min and centrifuged at 15,000 g and 4 °C for 20 min. Subsequently, part of the supernatant was diluted to a methanol concentration of 53% using liquid chromatography–mass spectrometry (LC–MS)-grade water. After centrifugation at 15,000 g and 4 °C for 20 min, the supernatant was injected into the LC–MS/MS system for analysis. Quality control (QC) samples were generated by pooling equal volumes from each experimental sample (three replicates) to assess the quality of metabolite extraction and detection.

### High-performance LC–MS/MS analysis

LC–MS/MS analysis was conducted by Novogene Co., Ltd. (Beijing, China), using an ExionLC™ AD high-performance liquid chromatograph (SCIEX) coupled with a QTRAP® 6500 + mass spectrometer (SCIEX). A linear gradient spanning 20 min was used for sample injection into an Xselect HSS T3 column (100 Å, 2.1 × 150 mm, 2.5 μm; column temperature: 50 °C) at a flow rate of 0.4 ml/min. The eluent consisted of eluent A (0.1% formic acid–water) and eluent B (0.1% formic acid–acetonitrile) [38]. The elution gradient was set as follows: 0 min, 98% A and 2% B; 2 min, 98% A and 2% B; 15 min, 0% A and 100% B; 17 min, 0% A and 100% B; 17.1 min, 98% A and 2% B; and 20 min, 98% A and 2% B. In the positive polarity mode of the QTRAP® 6500 + mass spectrometer, the settings were as follows: curtain gas: 35 psi; collision gas: medium; ionspray voltage: 5500 V; temperature: 550 °C; ion source gas: 1:60; and ion source gas: 2:60. In the negative polarity mode, the settings were as follows: curtain gas: 35 psi; collision gas: medium; ionspray voltage: − 4500 V; temperature: 550 °C; ion source gas: 1:60; and ion source gas: 2:60.

### Qualitative, quantitative, and QC analyses of metabolites

Using the Novogene database (novoDB, Beijing, China), we applied the multiple reaction monitoring (MRM) mode for the detection of *N. fuscus*, *L. brandtii*, and *M. musculus* experimental samples (Fig. S1). Quantitative analysis of metabolites was based on Q3 (product ion), whereas qualitative analysis of metabolites was based on Q1 (parent ion), Q3 (product ion), RT (retention time), DP (declustering voltage), and CE (collision energy). Data files generated via high-performance LC–MS/MS were imported into SCIEX OS Version 1.4 software for chromatographic peak integration and calibration. Chromatographic peak filter parameters were set as follows: minimum peak height: 500; signal/noise ratio: 5; and Gaussian smooth width: 1. The area of each chromatographic peak represented the relative content of the corresponding substance. All chromatographic peak area integral data were exported to obtain the qualitative and quantitative results for metabolites.

Metabolites change rapidly and are susceptible to disturbances from various factors, including instrument instability. Therefore, ensuring data QC during sample detection is crucial for result accuracy. Using QC data, we conducted total ion chromatogram (TIC) overlapping display analysis, Pearson correlation analysis, coefficient of variation (CV) distribution analysis, and principal component analysis (PCA) to evaluate data quality.

It is important to note that while the quasi-targeted metabolomics approach employed here allows for sensitive and accurate quantification of a broad spectrum of metabolites, it does not provide a fully comprehensive and unbiased overview of the entire metabolome. The metabolite panel was constructed based on the novoDB database, which encompasses a wide array of over 2200 compounds from human and animal sources as well as over 3250 compounds from plants. Therefore, our metabolite panel reflects the broad coverage of this database, aiming for a relatively unbiased profiling approach, rather than being selectively chosen to prioritize specific pathways of interest. It is important to acknowledge, however, that despite the extensive nature of this library, its predefined scope inherently imposes limitations on the comprehensiveness of our metabolomic coverage. Thus, our subsequent pathway enrichment analysis and the conclusions we derive are understood within the context of these boundaries.

### Metabolite data processing and analysis

Data processing and analysis were mainly performed using the Novomagic platform (https://magic.novogene.com/). Identified metabolites were annotated in the Kyoto Encyclopedia of Genes and Genomes (KEGG) database (https://www.genome.jp/kegg/), Human Metabolome Database (HMDB) database (https://www.hmdb.ca/), and Lipid Metabolites and Pathways Strategy (LIPID MAPS) database (https://www.lipidmaps.org/) to elucidate functional characteristics and classifications. PCA and partial least squares discriminant analysis (PLS-DA) were used for dimensionality reduction and regression analysis to distinguish overall metabolite differences. Permutation tests were performed to assess model quality. In addition, univariate analysis (t-test) was used to determine statistical significance. Differentially accumulated metabolites (DAMs) were defined as those with variable importance in projection (VIP) ≥ 1.0 and |log_2_FC|≥ 1.0 [39]. Among them, VIP is a weighted measure of the contribution of each metabolite to distinguishing groups. VIP ≥ 1 is considered an appropriate threshold for discriminant variables in the PLS-DA model [40]. To identify DAMs sharing similar metabolic patterns, we calculated correlations between DAMs using the cor function in R 4.3.2 (method: Pearson) [41] and constructed correlation network diagrams using the MetScape plug-in in Cytoscape 3.9.1 [42]. KEGG pathway enrichment analysis (based on hypergeometric test) of DAMs was used to determine their principal biological functions by using OmicShare (http://www.omicshare.com/tools). At the same time, considering the limitations of quasi-targeted metabolomics in the number of identifiable metabolites, we adopted two strategies to perform KEGG enrichment analysis: 1) using KEGG annotation results of all 721 detected metabolites as the background set; 2) using the R package massdatabase (https://massdatabase.tidymass.org/) to retrieve all metabolites involved in pathways of mice (5908 in total) within the KEGG database as the background set, in order to minimize bias in the pathway enrichment analysis of DAMs as much as possible.

### Integrated analysis of metabolomics and transcriptomics

In conjunction with prior transcriptome research [23], we reanalyzed differentially expressed genes (DEGs) (|Log_2_FoldChange|≥ 1 and *Padj* < 0.05) in the skeletal muscles of *N. fuscus*, *L. brandtii*, and *M. musculus* under hypoxic and normoxic conditions using DESeq2 (based on the Wald test and Benjamini–Hochberg multiple-testing correction) [43]. KEGG enrichment analysis and gene set enrichment analysis (GSEA), performed via the clusterProfiler package (version 4.10.1) in R [44, 45], revealed metabolic pathway changes among all unigenes. To explore skeletal muscle tissue’s hypoxia response mechanisms in these three species through integrated metabolomics and transcriptomics, we initially computed Pearson correlations between DAMs and DEGs. Subsequently, we simultaneously mapped DEGs and DAMs to the KEGG database to obtain shared pathway information. We then extracted correlation results for DAMs and DEGs in specific pathways and visualized these in correlation network diagrams using Cytoscape.

## Results

### Global metabolic profiling and QC

**Fig. 1:**
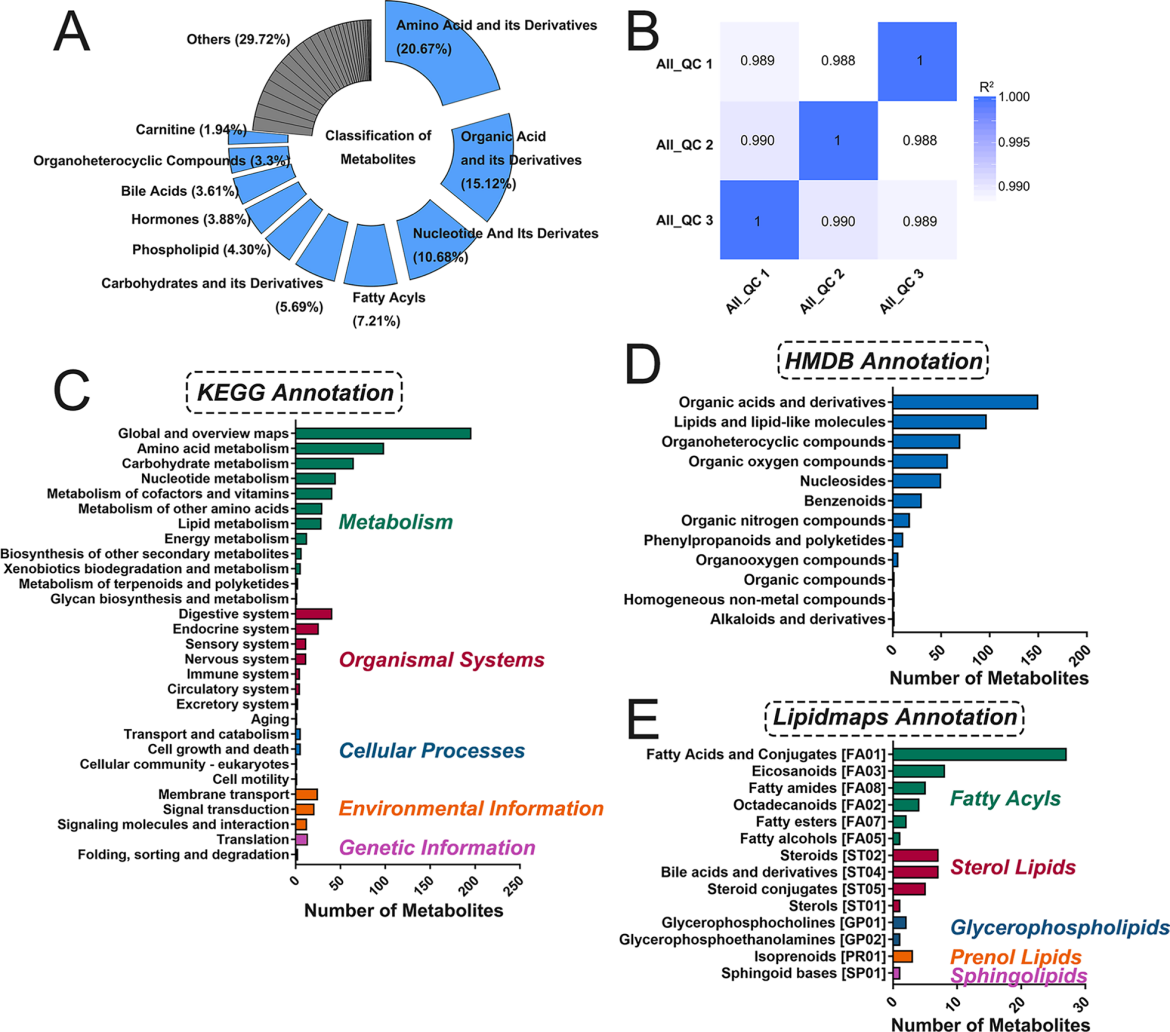
Qualitative and quality control (QC) analysis results for all metabolites in the skeletal muscle tissue of *Neodon fuscus*, *Lasiopodomys brandtii*, and *Mus musculus*. **A** Classification of all metabolites. **B** Correlation analysis results for three QC samples. **C** KEGG annotation and classification results for all metabolites. **D** HMDB annotation and classification results for all metabolites. **E** Lipidmaps annotation results and metabolite classification

We identified 721 metabolites across 38 categories in the skeletal muscle tissues of *N. fuscus*, *L. brandtii*, and *M. musculus* (Table S1). The top 10 categories of metabolites included 149 amino acids and their derivatives (20.67%), 109 organic acids and their derivatives (15.12%), 77 nucleotides and their derivatives (10.68%), 52 fatty acyls (7.21%), 41 carbohydrates and their derivatives (5.69%), 31 phospholipids (4.30%), 28 hormones (3.88%), 26 bile acids (3.61%), 24 organoheterocyclic compounds (3.30%), and 14 carnitines (1.94%) (Fig. 1A).

Regarding sample discrimination, the three QC samples exhibited strong correlations (*R*^2^ ≥ 0.989) (Fig. 1B). In addition, PCA successfully distinguished samples from different treatment groups (Fig. S2A), indicating robust data discrimination and consistency. Also, the median CV value of all identified metabolites across QC samples was 8.2591, and the proportion of metabolites with CV values less than 30% was 93.76%, indicating strong stability in the data detection process (Fig. S2B). Furthermore, TIC diagrams of the three QC samples, under both positive and negative ion modes, exhibited overlapping response intensities and retention times (Fig. S2C and D). These results collectively demonstrate stable sample detection processes and high data quality.

### Annotation results for metabolites

We obtained 343, 483, and 74 metabolite annotations in the KEGG, HMDB, and LIPID MAPS databases, respectively, and 38 metabolites were annotated in all three databases (Tables S2-S4). In detail, KEGG annotation results mainly including 5 categories: metabolism, organismal systems, cellular processes, environmental information, and genetic information (Fig. 1C; Table S2). In HMDB, metabolites were classified into 12 categories, with organic acids and derivatives, lipids and lipid-like molecules, and organoheterocyclic compounds ranking the highest (Fig. 1D; Table S3). LIPID MAPS, which focuses on lipid metabolism pathways, provided relatively fewer annotations. The primary annotation categories for metabolites in this study included fatty acyls, sterol lipids, glycerophospholipids, prenol lipids, and sphingolipids (Fig. 1E; Table S4).

### Screening and classification results for DAMs

**Fig. 2:**
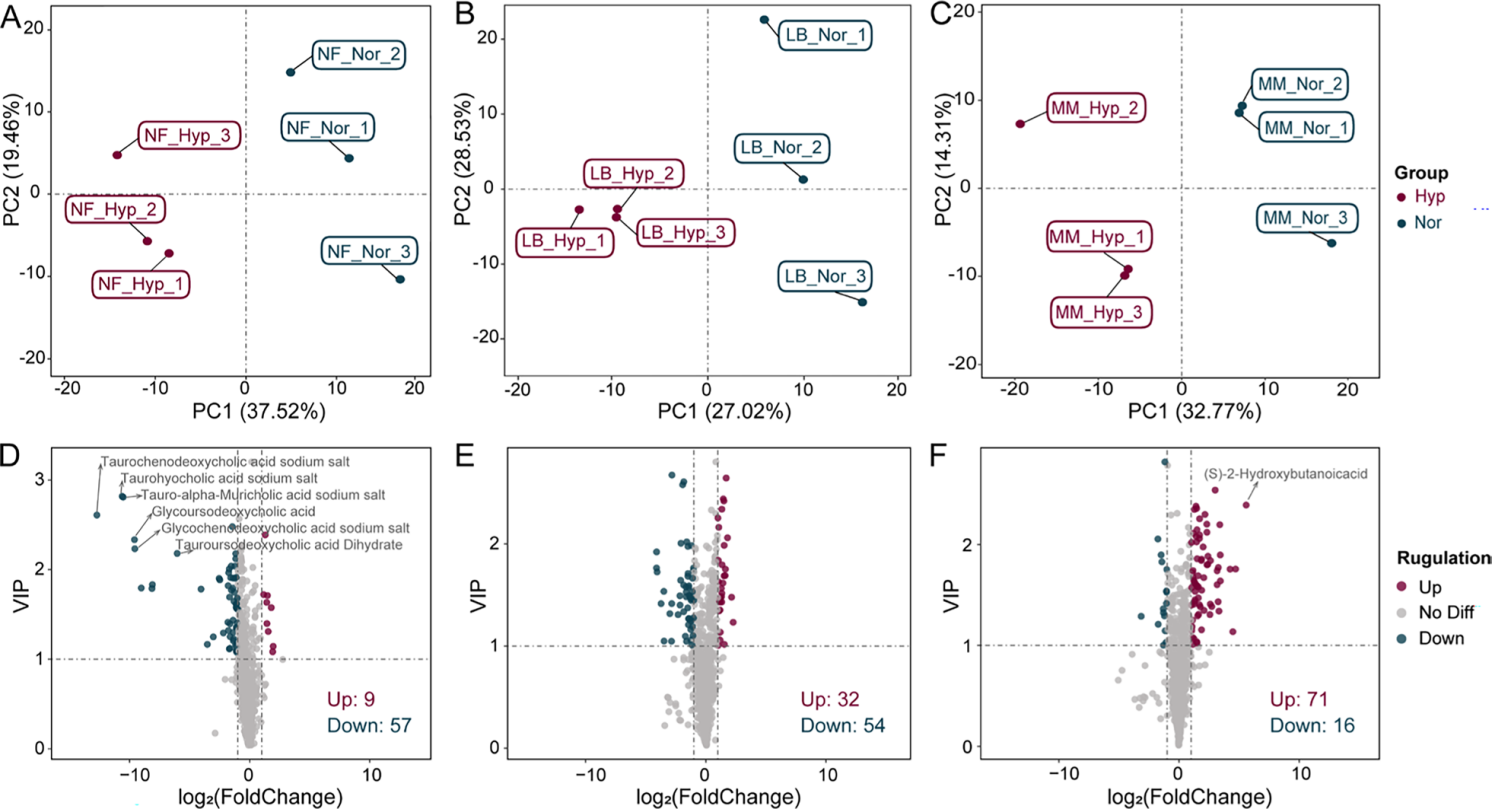
Screening results for differentially accumulated metabolites (DAMs). **A**–**C** PLS-DA results for *N. fuscus*, *L. brandtii*, and *M. musculus*. **D**–**F** Volcano plots for DAMs of *N. fuscus*, *L. brandtii*, and *M. musculus* (showing names of DAMs with | log_2_FC |> 5 and VIP > 2)

PLS-DA results displayed obvious sample separation among *N. fuscus* (56.98%, R2Y = 1.00, Q2Y = 0.43), *L. brandtii* (55.55%, R2Y = 0.99, Q2Y =  − 0.25), and *M. musculus* (47.08%, R2Y = 1.00, Q2Y = 0.13) samples under normoxia and hypoxia conditions (Fig. 2A–C). The R2/Q2 test results further substantiated the model’s reliability (Fig. S3A–C).

Regarding DAMs in the hypoxia/normoxia comparison, *N. fuscus* had 9 significantly upregulated and 57 significantly downregulated DAMs, *L. brandtii* had 32 significantly upregulated and 54 significantly downregulated DAMs, and *M. musculus* had 71 significantly upregulated and 16 significantly downregulated DAMs (Fig. 2D–F). Among all DAMs, organic acids and amino acids, involved in various metabolic pathways including glycolysis, exhibited the most significant fluctuations across the three species. Additionally, bile acids, carbohydrates, and fatty acyls showed prominent changes in *N. fuscus*, *L. brandtii*, and *M. musculus*, respectively (Fig. S4A–C; Table S5).

### Correlation analysis of DAMs

**Fig. 3:**
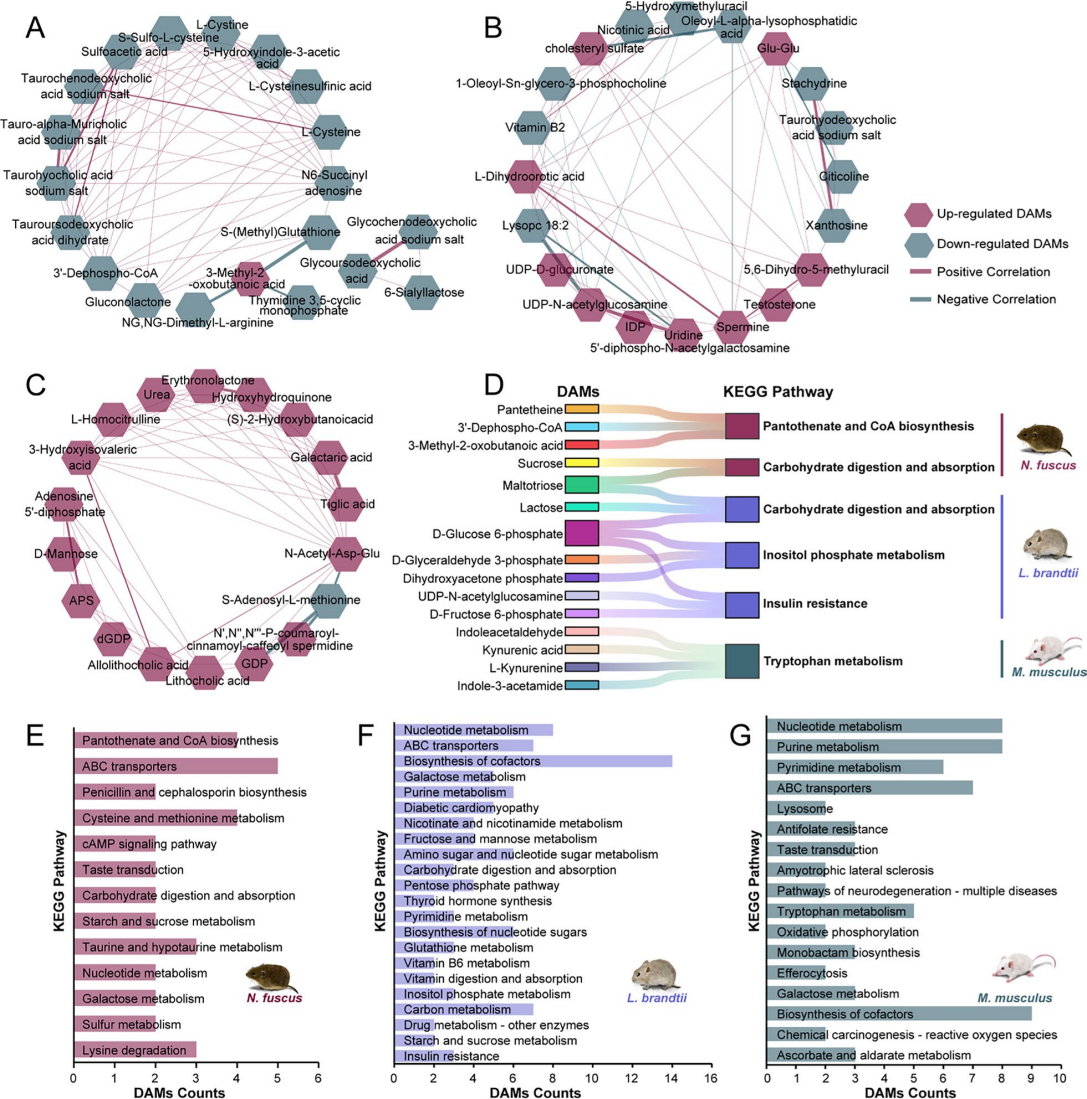
Correlation and pathway analysis results for DAMs. **A**–**C** Correlation analysis results for DAMs of *N. fuscus*, *L. brandtii*, and *M. musculus* (*R* > 0.8 and *P* < 0.05). Line thickness represents correlation strength. **D** KEGG enrichment results (using 721 detected metabolites as the background set) for DAMs of *N. fuscus*, *L. brandtii*, and *M. musculus* (*P* < 0.05). **E**–**G** KEGG enrichment results (using 5908 metabolites of mice from KEGG database as the background set) for DAMs of *N. fuscus*, *L. brandtii*, and *M. musculus* (*P* < 0.05)

Metabolites with similar expression patterns showed stronger correlations, potentially indicative of related biological functions [46]. In this study, we calculated Pearson correlations (retaining only results with *R* > 0.8 and *P* < 0.05) among the top 20 DAMs (sorted by VIP value) in each species. This analysis was conducted to initially investigate the variations in the response patterns of the primary DAMs under hypoxic conditions in the three species. For instance, in *N. fuscus*, significant positive correlations (*R* > 0.90) were observed among bile acids, such as taurohydroxylic acid sodium salt, tauro-alpha-muricholic acid sodium salt, taurochenodeoxylic acid sodium salt, and tauroursodeoxycholic acid dihydrate. Moreover, a strong positive correlation (*R* > 0.99) was observed between glycoursodeoxycholic acid and glycochenodeoxycholic acid sodium salt (Fig. 3A; Table S6). These bile acids have been associated with not only digestive functions but also extrahepatic signaling roles in regulating skeletal muscle oxygen and energy consumption via the G protein-coupled bile acid receptor 1 (TGR5), as well as promoting muscle cell differentiation [47, 48]. In *L. brandtii*, significant positive correlations were identified between cholesteryl sulfate and spermine (*R* = 0.94), as well as between spermine and UDP-N-acetylglucosamine (*R* = 0.83) (Fig. 3B; Table S6). These DAMs are known to possess antioxidant properties in vivo [49–51]. In *M. musculus*, positive correlations (*R* > 0.90) were observed among (S)-2-hydroxybutanoic acid, urea, and 3-hydroxyisovalic acid, which belong to organic acid and its derivatives (Fig. 3C; Table S6).

### KEGG enrichment results for DAMs

Using 721 detected metabolites as the background set, DAMs in *N. fuscus* and *L. brandtii* were significantly enriched in carbohydrate digestion and absorption (ko04973, *P* = 0.0428 for *N. fuscus* and *P* = 0.0073 for *L. brandtii*), which are related to glucose transport and metabolism. In *N. fuscus*, DAMs were also significantly enriched in pantothenate and CoA biosynthesis (ko00770, *P* = 0.0112), which is closely linked to glucose, lipid, and amino acid metabolism. Furthermore, DAMs in *L. brandtii* were significantly enriched in inositol phosphate metabolism (ko00562, *P* = 0.0166) and insulin resistance (ko04931, *P* = 0.0166). Most of these DAMs were intermediates of glycolysis, including D-glucose 6-phosphate, D-fructose 6-phosphate, dihydroxyacetone phosphate (DHAP), and D-glyceraldehyde 3-phosphate. In *M. musculus*, DAMs were significantly enriched in tryptophan metabolism, with kynurenic acid playing a role in scavenging free radicals and reducing cell damage [52] (Fig. 3D; Table S7).

Employing a background set of 5908 metabolites of mice from the KEGG database, and building upon the comprehensive coverage of the aforementioned enrichment results, DAMs in *N. fuscus*, *L. brandtii*, and *M. musculus* exhibited significant enrichment in 13, 22, and 17 pathways, respectively (Fig. 3E-G; Table S7). This approach yielded more systematic and less biased outcomes. In *N. fuscus*, DAMs were significantly enriched in the lysine degradation (ko00310, *P* = 0.0460), where the product succinic acid may enter the TCA cycle and contribute to energy metabolism. Concurrently, DAMs were also enriched in the cysteine and methionine metabolism (ko00270, *P* = 0.0038) and taurine and hypotaurine metabolism (ko00430, *P* = 0.0179) pathways, potentially influencing the regulation of pyruvate synthesis. In *L. brandtii*, DAMs were significantly enriched in glutathione metabolism (ko00480, *P* = 0.0232) related to antioxidant function, as well as pentose phosphate pathway (ko00030, *P* = 0.0035) and nicotine and nicotinamide metabolism (ko00760, *P* = 0.0008) involved in glucose oxidation. In *M. musculus*, DAMs were also significantly enriched in ascorbate and aldarate metabolism (ko00053, *P* = 0.0480) related to antioxidant function, as well as the oxidative phosphorylation (ko00190, *P* = 0.0066) related to energy generation. These new enriched pathways provide a more systematic basis for further investigation into the hypoxia response mechanisms across these three voles.

### Transcriptomics analysis results

Transcriptome sequencing data for skeletal muscles of *N. fuscus*, *L. brandtii*, and *M. musculus* under normoxic and hypoxic conditions are available in the NCBI database (Bioproject ID: PRJNA993829). We employed DESeq2 for reanalysis of previously published results [23], as it demonstrated superior performance (in terms of coverage and fold change results) in identifying DEGs compared with edgeR based on previous studies on eukaryotic organisms, such as humans and mice, as well as RT-qPCR experimental data [53, 54]. Consequently, we identified 1523 DEGs in *N. fuscus* (601 upregulated and 922 downregulated), 1978 DEGs in *L. brandtii* (1784 upregulated and 194 downregulated), and 1683 DEGs in *M. musculus* (1011 upregulated and 672 downregulated). This analysis included > 99% of the genes identified via edgeR. After filtering out genes with substantial differences based on annotation results from Nr, Swiss-Prot, EggNOG, and KOBAS databases, we obtained 221 DEGs in *N. fuscus* (80 upregulated and 141 downregulated), 243 DEGs in *L. brandtii* (204 upregulated and 39 downregulated), and 326 DEGs in *M. musculus* (151 upregulated and 175 downregulated) (Table S8).

**Fig. 4:**
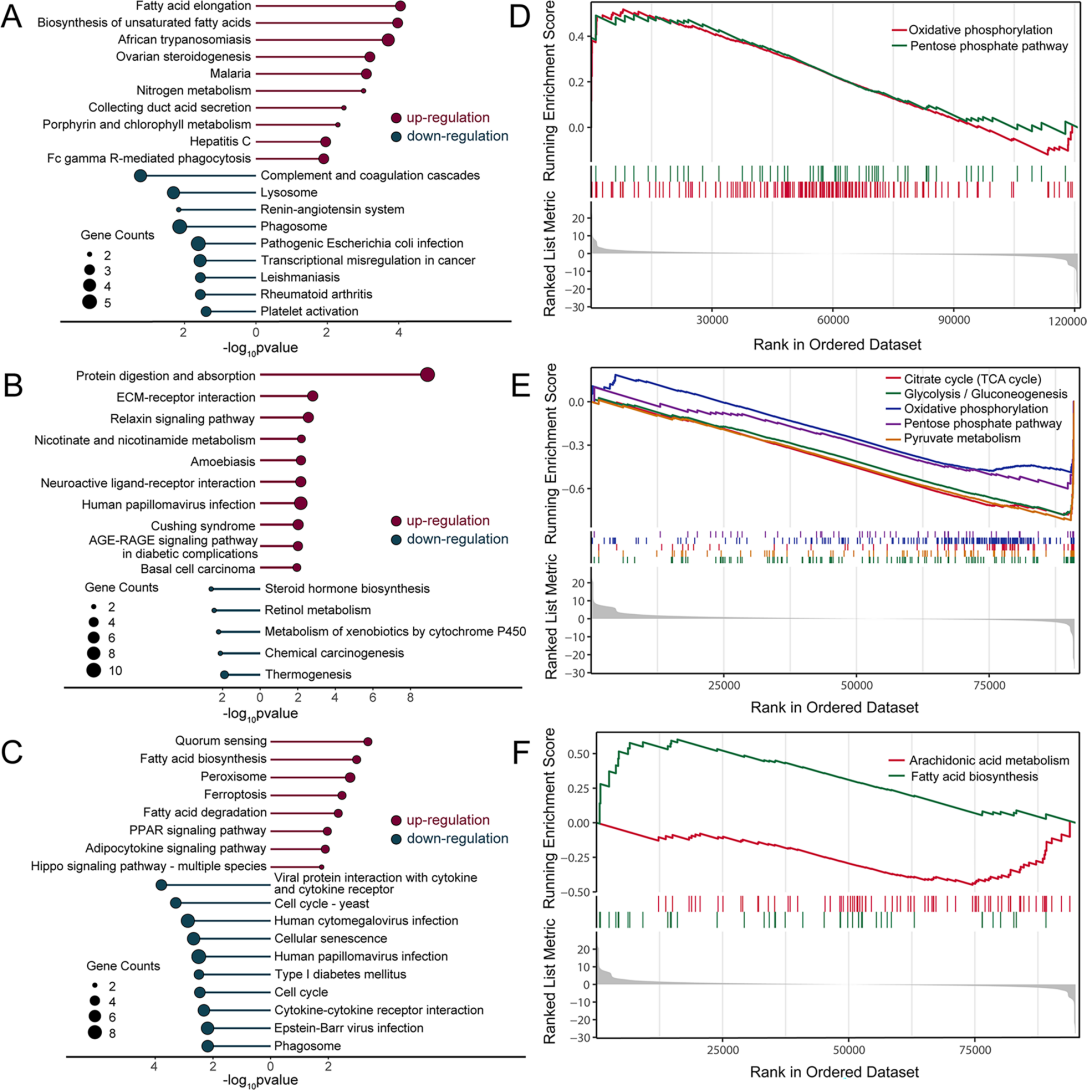
Transcriptome pathway analysis results. **A**–**C** KEGG enrichment results for differentially expressed genes (DEGs) from *N. fuscus*, *L. brandtii*, and *M. musculus*. The top 10 KEGG pathways of upregulated or downregulated DEGs are shown. **D**, **E**, **F** KEGG pathways related to energy metabolism based on gene set enrichment analysis (GSEA) results for all unigenes of *N. fuscus*, *L. brandtii*, and *M. musculus*

Significant KEGG pathways (*P* < 0.05 and ≥ 2 DEGs) were identified for each species. Upregulated DEGs in *N. fuscus* were significantly enriched in 12 KEGG pathways, including African trypanosomiasis (ko05143), malaria (ko05144), and porphyrin and chlorophyll metabolism (ko00860). In these pathways, various DEGs, such as *Hba*, *Hbb*, *Alas2*, and *Uros*, were associated with heme and hemoglobin syntheses, thereby potentially enhancing oxygen transport ability of *N. fuscus* under hypoxic conditions. Downregulated DEGs in *N. fuscus* were enriched in nine KEGG pathways, including complement and coagulation cascades (ko04610), platelet activation (ko04611), and pathogenic *Escherichia coli* infection (ko05130) (Fig. 4A; Table S9).

In *L. brandtii*, upregulated DEGs were significantly enriched in 25 KEGG pathways. The nicotinate and nicotinamide metabolism (ko00760) pathway included the DEGs *Bst1*, *Nmnat2*, and *Nmrk2*, which play roles in beta-nicotinamide adenine dinucleotide phosphate (NADP^+^) production. Downregulated DEGs were significantly enriched in 5 KEGG pathways, including thermogenesis (ko04714) associated with the DEGs *Cpt2* and *Pnpla2*, which are involved in lipid decomposition and fatty acid β-oxidation. Their downregulation indicates that the energy generated through fatty acid oxidation in *L. brandtii* may be diminished in a hypoxic environment (Fig. 4B; Table S9).

Upregulated DEGs in *M. musculus* were significantly enriched in eight KEGG pathways, with upregulated *Ascl6*, which hinders fatty acid β-oxidation, appearing in most pathways. Downregulated DEGs in *M. musculus* were significantly enriched in 32 KEGG pathways related to cell activity and diseases, such as cellular senescence (ko04218), cell cycle (ko04110), human cytomegalovirus infection (ko05163), and human papillomavirus infection (ko05165) (Fig. 4C; Table S9).

Using GSEA and KEGG annotation results as backgrounds, we assessed the trend of changes in all genes related to metabolism. Significantly enriched KEGG pathways were identified based on the following criteria: |Normalized Enrichment Score (NES)|> 1, *P* < 0.05, and *Padj* < 0.25. *N. fuscus* exhibited 52 significantly enriched KEGG pathways, with oxidative phosphorylation (NES = 1.8932) and the pentose phosphate pathway (NES = 1.4962) being related to energy metabolism. Core genes involved in these pathways, such as *Mt-Nd1*, *Mt-Nd4*, *Ndufb7*, *Mt-Co1*, *Mt-Co2*, *Atp6*, *Atpa*, *Aldoa*, *Rgn*, and *Pgls*, indicated upregulation of oxidative phosphorylation and the pentose phosphate pathway under hypoxic conditions (Fig. 4D; Table S10).

*Lasiopodomys brandtii* exhibited 102 significantly enriched KEGG pathways, including glycolysis/gluconeogenesis (NES =  − 2.6782), pyruvate metabolism (NES =  − 2.6102), tricarboxylic acid (TCA) cycle (NES =  − 2.4596), oxidative phosphorylation (NES =  − 1.7932), and the pentose phosphate pathway (NES =  − 1.7533) related to energy metabolism. Core genes, such as *Mt-Nd1*, *Mt-Nd4*, *Ndufa3*, *Ndufa5*, *Mt-Co1*, *Mt-Co2*, *Atp4a*, and *Atp6*, indicated inhibition of oxidative phosphorylation under hypoxic conditions. In addition, changes in core genes, such as *Aldob*, *Pck1*, and *Ogdhl*, indicated downregulation of certain intermediate reactions in glycolysis/gluconeogenesis and the TCA cycle in *L. brandtii* (Fig. 4E; Table S10).

*Mus musculus* exhibited 68 significantly enriched KEGG pathways. Fatty acid biosynthesis (NES = 1.5942) and arachidonic acid metabolism (NES =  − 1.4943) were related to energy metabolism. Core genes, including *Acacb* and *Acsl6*, indicated that fatty acid β-oxidation was inhibited under hypoxia. Changes in core genes, such as *Alox5*, *Alox12*, *Cyp2u1*, *Cyp4f6*, *Pla2g4a*, and *Pla2g4e*, indicated reduced release of arachidonic acid from glycerol phospholipids and altered metabolism of arachidonic acid under hypoxia (Fig. 4F; Table S10).

### KEGG pathways shared by DEGs and DAMs

**Fig. 5:**
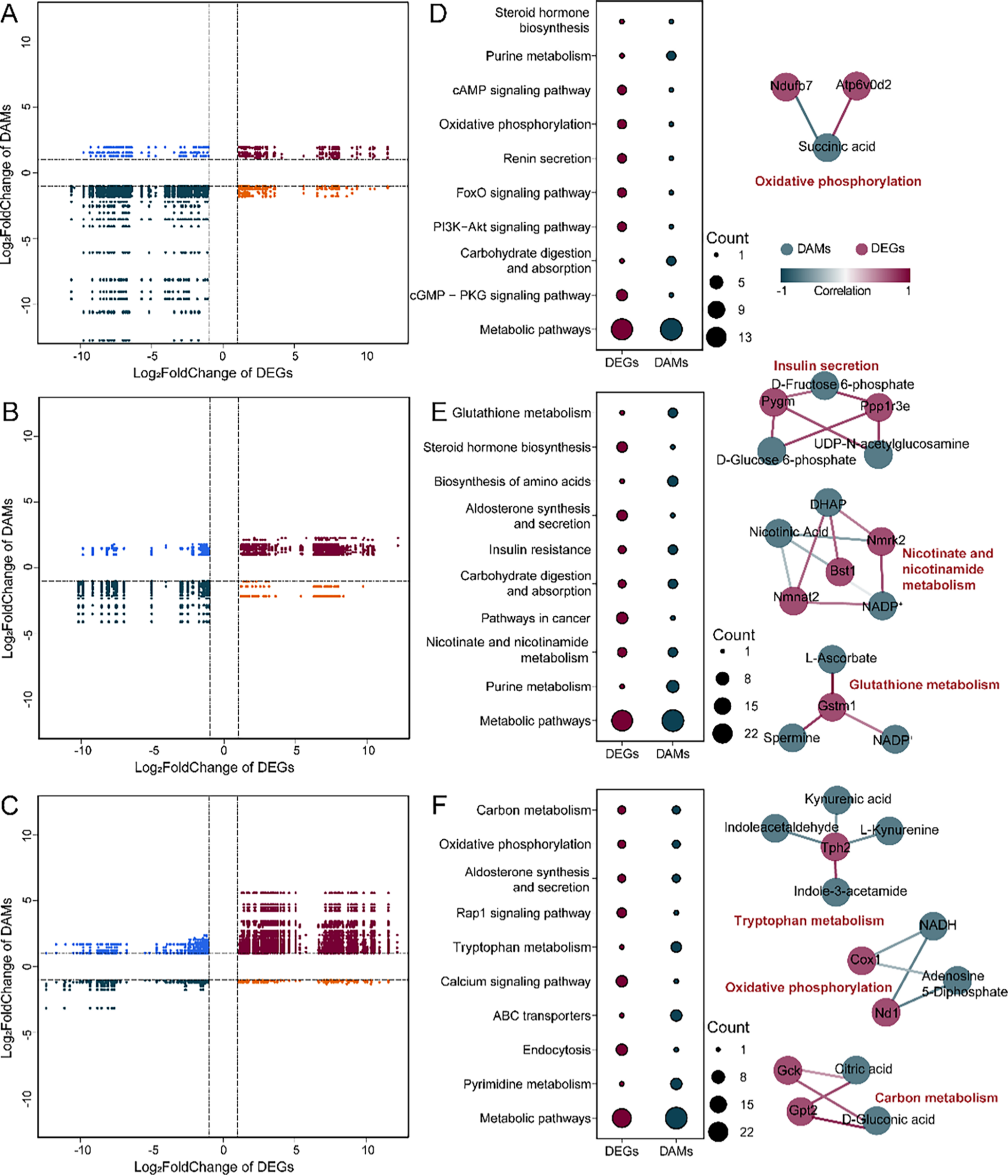
Integrated metabolomics and transcriptomics analysis results. **A**–**C** Results of Pearson correlation analysis (*R* > 0.8 and *P* < 0.05) between DEGs and DAMs for *N. fuscus*, *L. brandtii*, and *M. musculus*. **D**–**F** KEGG pathways shared by DEGs and DAMs (10 pathways with the highest number of DEGs and DAMs are shown), and correlation network diagrams for DEGs and DAMs in some pathways

After identifying significant correlations (*R* > 0.8 and *P* < 0.05), we found that positive correlations between DAMs and DEGs were more prevalent than negative correlations in all the species we studied (Fig. 5A–C; Table S11). Exploring pathways shared by DEGs and DAMs revealed differences related to glucose metabolism, oxidative phosphorylation, and antioxidant activity across the three species. In *N. fuscus*, 11 shared pathways were identified, with oxidative phosphorylation (ko00190) being a key pathway in biological aerobic respiration to generate energy. DEGs and DAMs involved in this pathway included *Ndufb7*, *Atp6v0d2*, and succinic acid (Fig. 5D; Table S12).

In *L. brandtii*, 27 shared pathways were identified. Notably, glutathione metabolism (ko00480) was related to biological antioxidant defense mechanisms, featuring various DEGs and DAMs, such as *Gstm1*, L-ascorbate, and spermine. The nicotinate and nicotinamide metabolism (ko00760) pathway contributed to nicotinamide adenine dinucleotide (NAD^+^), NADP^+^, and other coenzyme production, including several DEGs and DAMs, such as *Bst1*, *Nmnat2*, *Nmrk2*, NADP^+^, and DHAP. Insulin resistance (ko04931) is closely related to glucose metabolism and was associated with certain DEGs and DAMs, such as *Pygm*, *Ppp1r3e*, D-glucose-6-phosphate, D-fructose-6-phosphate, and UDP-N-acetylglucosamine (Fig. 5E; Table S12).

In *M. musculus*, 32 shared pathways were identified. The DEGs and DAMs involved in oxidative phosphorylation (ko00190) included *Nd1*, NADH, and adenosine 5'-diphosphate. Carbon metabolism (ko01200) is related to glycolysis, the TCA cycle, and other biochemical reactions, and it was associated with the DEGs and DAMs *Gck*, *Gpt2*, citric acid, and D-glucuronic acid, indicating changes in these processes (Fig. 5F; Table S12).

## Discussion

Skeletal muscle uses various substrates, including pyruvate, fatty acids, amino acids, and others, to generate adenosine triphosphate (ATP) through the TCA cycle, fatty acid β-oxidation, and oxidative phosphorylation. These processes provide the primary energy source for muscle activities. In addition, a small amount of ATP is produced through glycolysis in the cytoplasm [55–57]. Although glucose is the preferred substrate for energy generation in skeletal muscle, during periods of high energy demand, fatty acid β-oxidation serves as an efficient alternative mode of energy generation [58, 59]. Typically, nonhypoxic-tolerant species compensate for energy deficiency in hypoxic environments by enhancing anaerobic glycolysis caused by limited oxygen supply [24–26]. Conversely, hypoxic-tolerant species exhibit enhanced oxygen transport capabilities under hypoxic conditions [28–30]. These differences can result in distinct metabolic patterns in glucose and fatty acid utilization. Notably, significant variations in glycolysis, fatty acid oxidation, and oxidative phosphorylation patterns were found among *N. fuscus*, *L. brandtii*, and *M. musculus* in the present study.

**Fig. 6:**
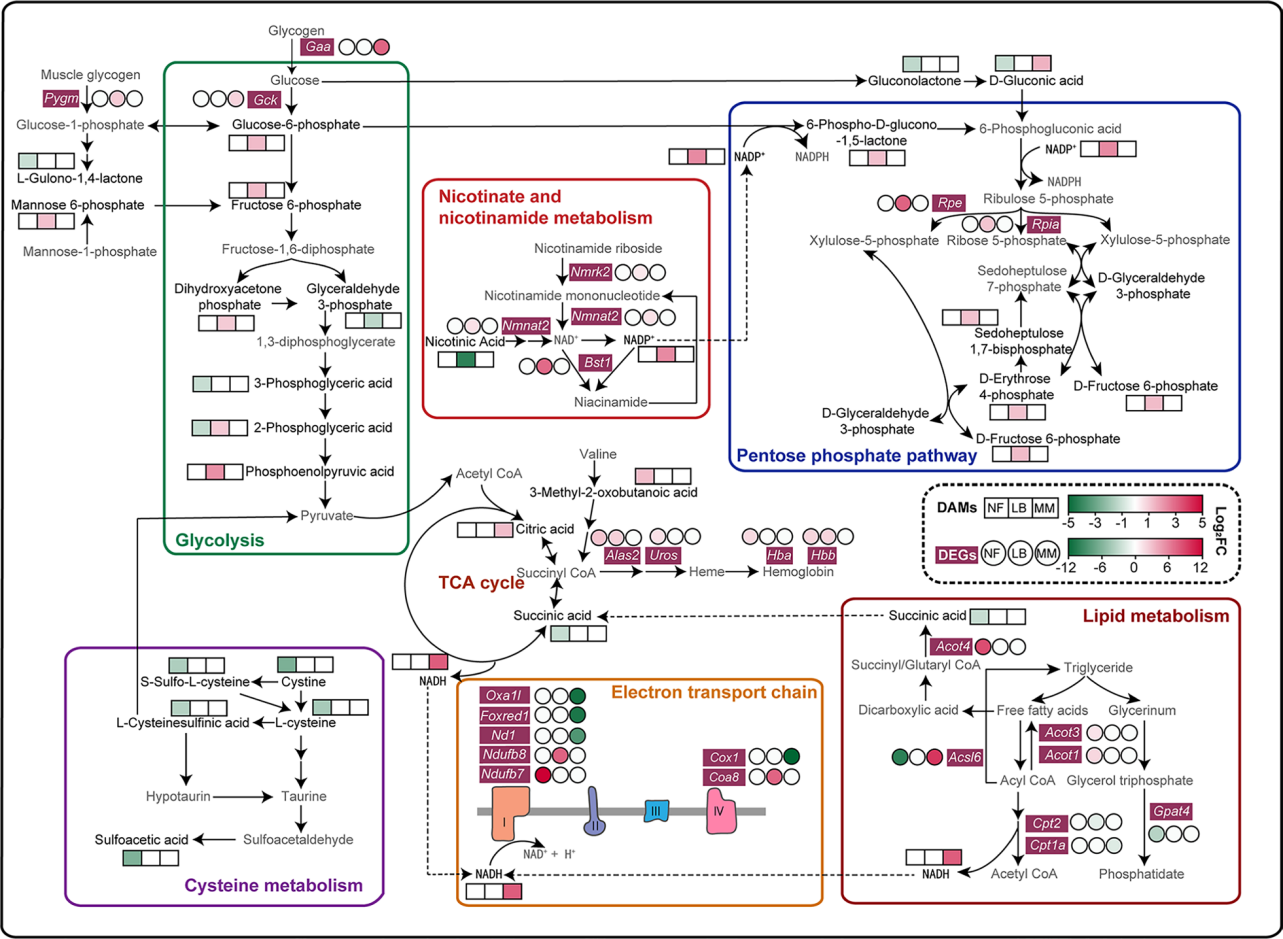
Regulation network of DAMs and DEGs in the skeletal muscle of *N. fuscus*, *L. brandtii*, and *M. musculus.* NF: *N. fuscus*; LB: *L. brandtii*; MM: *M. musculus*. DAMs are shown in black, whereas other metabolites are shown in gray. DEGs are shown in purple–red squares. Black and dashed arrows represent the directions of the biochemical reaction and metabolite transfer, respectively

Regarding *N. fuscus*, several glycolysis and glucose metabolism pathway metabolites were downregulated under hypoxic conditions, including 3-phosphoglyceric acid, 2-phosphoglyceric acid, gluconolactone, D-gluconic acid, and L-gulono-1,4-lactone. This downregulation may suggest reduced glucose utilization in *N. fuscus* under hypoxia compared with normoxic conditions. Furthermore, as sources of pyruvate [60], many metabolites in the cysteine metabolism pathway, such as L-cysteine, cystine, S-sulfo-L-cysteine, L-cysteinesulfinic acid, and sulfoacetic acid, were also downregulated, may further limiting pyruvate availability for glycolysis. Conversely, the upregulation of 3-methyl-2-oxobutanoic acid in *N. fuscus* potentially lead to the production of succinyl CoA, thereby participating in the TCA cycle [61]. Moreover, previous transcriptomics research revealed that specific DEGs, including *Acsl6*, *Gpat4*, *Aoct4*, and *Ndufb7*, promote fatty acid β-oxidation and electron transport chain conduction in *N. fuscus* skeletal muscle, thereby sustaining energy production [23]. This finding is supported by our GSEA results, which indicated significant enhancement in oxidative phosphorylation (Fig. 4D). Consequently, these results suggest that *N. fuscus* relies primarily on fatty acids as an energy source, rather than glucose, in hypoxic environments (Fig. 6).

In *L. brandtii*, analysis of shared KEGG pathways revealed the involvement of *Pygm*, D-glucose 6-phosphate, and D-fructose 6-phosphate in the insulin secretion pathway. Among these, *Pygm*, upregulated in this context, encodes muscle glycogen phosphorylase, a key enzyme initiating the first step in glycogenolysis [62]. The upregulation of glucose-6-phosphate and fructose-6-phosphate, both of which are intermediate products of glycolysis, correspondingly aligned with our metabolomic findings, which indicated the upregulation of other DAMs as intermediate products of glycolysis, including DHAP, 2-phosphoglyceric acid, and phosphoenolpyruvic acid. These results suggest that under hypoxic conditions, the skeletal muscle of *L. brandtii* may rely on glycolysis to produce energy. Similar to *N. fuscus*, exposure of *L. brandtii* to hypoxia led to the upregulation of genes related to the electron transport chain, including *Ndufb8* and *Coa8*. *Ndufb8*, which encodes the subunit of complex I, plays an important role in maintaining the stability and activity of complex I [63]. In contrast, *Coa8*, which encodes the cytochrome C oxidase assembly factor 8, participates in the assembly of complex IV and protects complex IV from oxidative stress–induced degradation [64]. Consequently, oxidative phosphorylation in the skeletal muscle of *L. brandtii* may depend on the glycolysis pathway in hypoxic environments.

In addition, the pentose phosphate pathway, an alternative route for glucose oxidation, underwent significant changes in response to hypoxia in *L. brandtii*. The upregulation of 6-phospho-D-glucono-1,5-lactone, an intermediate product in the oxidative stage, alongside intermediate products, such as sedoheptulose 1,7-bisphosphate and D-erythrose 4-phosphate, in the nonoxidative stage. This finding aligns with our transcriptome analysis, which also revealed upregulation of *Rpia* and *Rpe*. *Rpia* encodes ribose 5-phosphate isomerase, facilitating the conversion of ribulose 5-phosphate to ribose 5-phosphate in the nonoxidative stage of the pentose phosphate pathway [65]. Conversely, *Rpe* promotes the conversion of ribulose 5-phosphate to xylulose-5-phosphate [66]. The pentose phosphate pathway not only produces ribose 5-phosphate for nucleotide biosynthesis but also generates NADPH, which is critical for maintaining cellular glutathione in its reduced state and an essential component of antioxidant defense [67]. Notably, shared KEGG pathway analysis indicated the participation of *Bst1*, *Nmnat2*, *Nmrk2*, and NADP^+^ in the nicotinate and nicotinamide metabolism pathway (ko00760) (Fig. 5E). Upregulated *Nmnat2* and *Nmrk2* primarily synthesize NAD^+^ and NADP^+^ through the mammalian salvage pathway [68, 69]. Conversely, the downregulation of nicotinic acid suggests a reduced contribution to NAD^+^ synthesis via the Preiss–Handler pathway [70]. These findings indicate that *L. brandtii* may accumulate NADP^+^ through the regulation of nicotinate and nicotinamide metabolism in hypoxic environments, thereby supporting the progression of the pentose phosphate pathway (Fig. 6).

Regarding *M. musculus*, the upregulation of *Gaa*, encoding α-glucosidase, and *Gck*, encoding hexokinase, suggests enhancement of glycolysis under hypoxic conditions [71, 72]. Meanwhile, hypoxia negatively impacts fatty acid β-oxidation in the skeletal muscle of *M. musculus*. In addition to the previously discovered upregulation of *Acsl6*, which can reduce the ability of fatty acid β-oxidation [23], we observed the downregulation of *Cpt1a*, which encodes a rate-limiting enzyme for fatty acid transport in the mitochondria. Notably, *Cpt1a* can be inhibited by hypoxia-inducible factor (HIF) to reduce fatty acid β-oxidation [73].

In contrast to *N. fuscus* and *L. brandtii*, *M. musculus* exhibited downregulation of several genes involved in the electron transport chain, including *Nd1*, *Foxred1*, *Oxa1l*, and *Cox1*. Of these, *Nd1*, encoded by mitochondrial DNA, is a central component of complex I in the electron transfer chain [74]. *Foxred1*, which encodes the FAD-dependent oxidoreductase domain protein 1, participates in the mid-to-late stages of complex I assembly [75]. *Oxa1l* encodes a mitochondrial inner membrane protein that is essential for complex I and ATP synthase biosynthesis in the mitochondria [76]. *Cox1* encodes cytochrome C oxidase subunit 1, which is the core subunit of cytochrome C oxidase (complex IV) and is highly dependent on oxygen [77, 78]. These findings indicate that the oxidative phosphorylation level in *M. musculus* decreases under hypoxic conditions, thereby resulting in a decrease in ATP synthesis. Importantly, the usual oxidative phosphorylation process relies on NADH for electron transfer. However, we observed a significant upregulation of NADH expression in the skeletal muscle of *M. musculus* under hypoxic conditions. Yang et al. [79] demonstrated that NADH accumulation occurs under hypoxic conditions or when oxygen utilization is ineffective, directly resulting from electron transport chain impairment. Furthermore, Liu et al. [80] indicated that NADH accumulation inhibits isocitrate dehydrogenase, thereby impeding the TCA cycle. Consequently, owing to the inhibited fatty acid β-oxidation, TCA cycle, and oxidative phosphorylation, the skeletal muscle of *M. musculus* likely relies more on the glycolysis pathway for energy generation under hypoxic conditions (Fig. 6).

In general, *N. fuscus*, adapted to high-altitude environments, appears likely to sustain energy supply through regulated fatty acid oxidation under low-oxygen conditions. In contrast, *L. brandtii* and *M. musculus*, distributed in mid- and low-altitude regions, respectively, seem to rely on aerobic oxidation and anaerobic glycolysis of glucose, respectively, for energy maintenance in low-oxygen environments. Although this species-specific metabolic pattern under hypoxia warrants further experimental verification, previous studies on other plateau-dwelling species, such as high-altitude deer mice (*Peromyscus maniculatus*), have also indicated enhanced fatty acid oxidation under hypoxic conditions [81]. Consequently, we hypothesize that regulated fatty acid oxidation might constitute a distinctive metabolic pattern in the skeletal muscle tissue of plateau-dwelling animals, enabling them to tolerate hypoxic environments.

Skeletal muscles are highly dependent on oxygen supply to maintain energy production and redox homeostasis [22, 23], while *N. fuscus*, *L. brandtii*, and *M. musculus* exhibit different oxygen transport strategies under hypoxia environments. Based on transcriptomic analysis, we found that *N. fuscus* significantly enriched in porphyrin and chlorophyll metabolism, African trypanosomiasis and malaria pathways, containing multiple upregulated genes involved in hemoglobin synthesis, such as *Alas2*, *Uros*, *Hba*, and *Hbb* (Fig. 4A). Among them, the δ-aminolevulinic acid synthase 2 encoded by *Alas2* is the rate limiting enzyme for heme synthesis and plays an important role in regulating iron and heme homeostasis [82]; Uroporphyrin III synthase encoded by *Uros* is the fourth enzyme involved in heme synthesis and serves as the central hub of heme synthesis [83]; *Hba* and *Hbb* are involved in encoding hemoglobin subunits [84]. The upregulation of these genes may have a potential connection with the induction of HIF-1α [85, 86], thereby helping to enhance the oxygen transport capacity of *N. fuscus* under hypoxia environments. Similar to the *N. fuscus*, *Alas2* and *Hbb* were also found to be upregulated in *L. brandtii*, demonstrating a certain oxygen transport capacity. Contrarily, we found non DEGs related to oxygen transport in *M. musculus*, which further suggests the unique strategy of low oxygen tolerant species (Fig. 6).

**Fig. 7:**
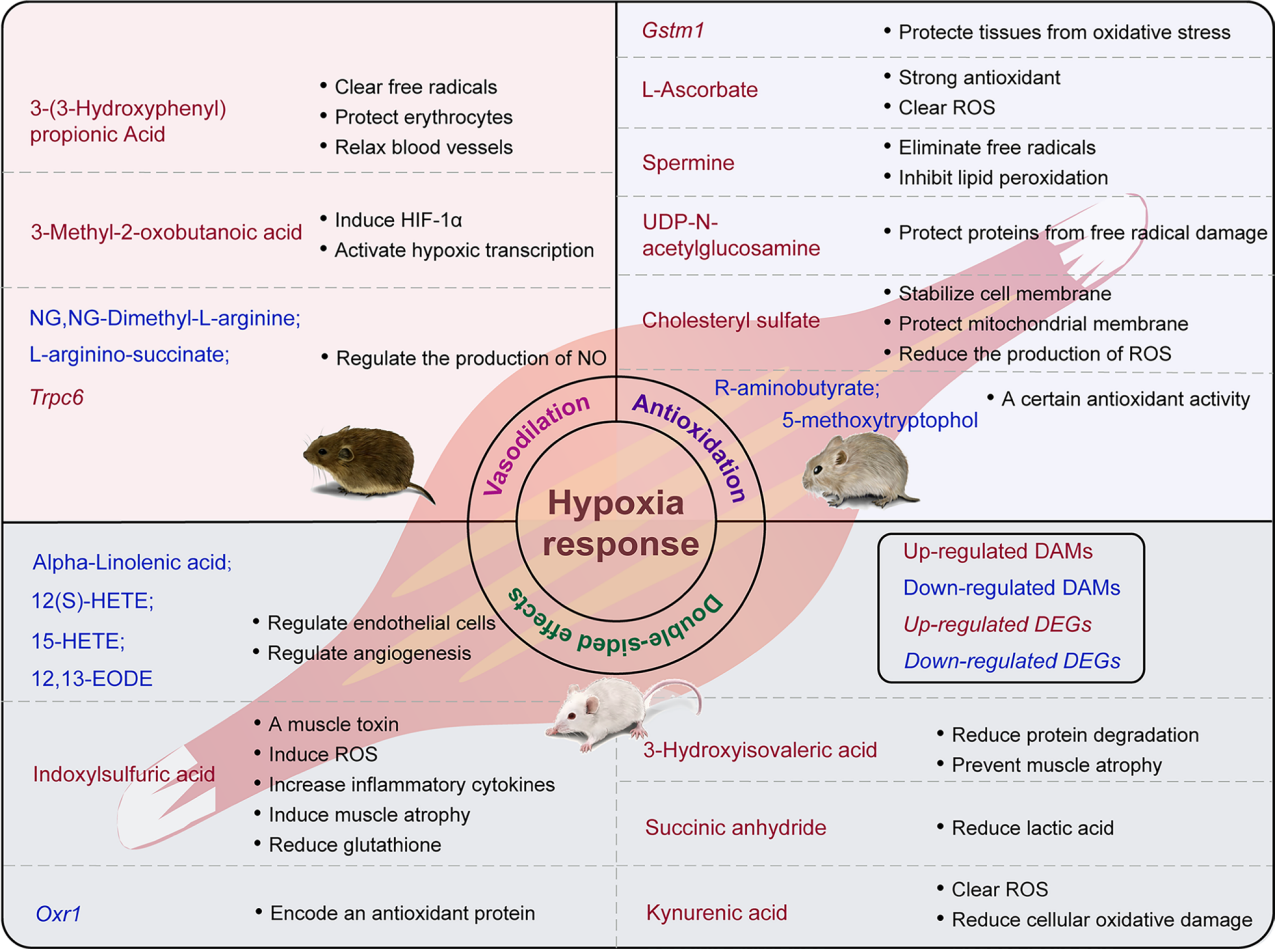
Differences in DAMs and DEGs in hypoxic adaptation mechanisms, such as vasodilation, antioxidation, and cell damage, among *N. fuscus*, *L. brandtii*, and *M. musculus*

In addition to exhibiting different metabolic pattern preferences and oxygen transport capacity under hypoxic conditions, the three rodent species exhibited species-specific responses in blood vessel relaxation, antioxidant defense, anti-inflammatory processes, and other aspects. Hypoxia is an important factor contributing to systemic hypertension and vascular diseases [87]. By screening DEGs and DAMs, we identified various ways in which the skeletal muscle of *N. fuscus* relaxed blood vessels, which proved advantageous for oxygen transport. First, the upregulation of 3-(3-hydroxyphenyl) propionic acid played a pivotal role in reducing arterial blood pressure and dilating blood vessels [88]. 3-(3-hydroxyphenyl) also possesses antioxidant properties, removing free radicals and protecting red blood cells from oxidative damage [89]. In addition, hypoxic exposure led to a decrease in the expression of NG, NG-dimethyl-L-arginine in the skeletal muscle of *N. fuscus*. Endogenous NG, NG-dimethyl-L-arginine can suppress nitric oxide (NO) production by inhibiting nitric oxide synthase [90]. NO is a crucial signaling molecule in the body, regulating vasodilation, nerve conduction, and immune function [91, 92]. Therefore, the downregulation of NG, NG-dimethyl-L-arginine was conducive to vasodilation. Notably, correlation analysis of DAMs (Fig. 3A) revealed a significant negative correlation between NG, NG-dimethyl-L-arginine and 3-methyl-2-oxobutanoic acid (*R* =  − 0.88). It has been observed that 3-methyl-2-oxobutanoic acid can inhibit the activity of HIF-1α proline hydroxylase, inducing HIF-1α accumulation and activating transcription processes associated with hypoxia adaptation [93]. In addition, L-arginino-succinate inhibits NO synthase [94]. Furthermore, its accumulation in the brain reduces glutathione levels, leading to protein oxidative damage and reactive oxygen species (ROS) production and ultimately diminishing the brain’s antioxidant capacity [95]. The decreased expression of L-arginino-succinate in the skeletal muscle of *N. fuscus* under hypoxic conditions aligned with the change in NG and NG-dimethyl-L-arginine expression. Similarly, we found that the expression of *Trpc6*, a member of the TRPC channel family, was upregulated in our transcriptome analysis of *N. fuscus*. Research on human pulmonary artery smooth muscle cells has demonstrated that the upregulation of *Trpc6* is related to the increase in Ca^2+^ levels induced by hypoxia [96]. This elevation in Ca^2+^ levels enhances NO production, promoting blood vessel relaxation [97] (Fig. 7).

Organisms’ increased production of ROS is closely linked to oxidative stress and the development of numerous chronic diseases. In the present study, we observed enhanced antioxidant defense mechanisms in the skeletal muscle of *L. brandtii* under hypoxic conditions. Our analysis of shared KEGG pathways in *L. brandtii* identified *Gstm1*, L-ascorbate, and spermine involvement in glutathione metabolism. L-ascorbate, commonly known as vitamin C, is a well-established antioxidant synthesized in most vertebrates, except primates and guinea pigs. It effectively scavenges ROS [98]. Furthermore, the accumulation of L-ascorbate in muscle tissue has been shown to protect muscle cells from oxidative stress and is essential for skeletal muscle structure and function [99]. Spermine serves as a natural antioxidant and anti-inflammatory agent, eliminating free radicals, inhibiting tissue lipid peroxidation, and protecting cells from oxidative damage [50]. *Gstm1* encodes glutathione-S-transferase, facilitating the binding of glutathione with ROS or exogenous substances, thereby safeguarding tissues from oxidative stress or toxic damage [100]. Therefore, the upregulation of *Gstm1*, L-ascorbate, and spermine expression in the skeletal muscle of *L. brandtii* significantly contributed to its antioxidant defense. In addition, through metabolomics analysis, we identified other DAMs with antioxidant properties in *L. brandtii*, such as upregulated UDP-N-acetylglucosamine and cholesteryl sulfate and downregulated R-aminobutyrate and 5-methoxytryptophol. Among these, UDP-N-acetylglucosamine is the end product of the hexosamine metabolism pathway and protects proteins from free radicals by participating in glycosylation activation [49]. Cholesteryl sulfate plays a vital role in stabilizing cell membranes, reducing mitochondrial membrane potential collapse, and limiting ROS production [51]. Moreover, R-aminobutyrate and 5-methoxytryptophol have been reported to exhibit antioxidant and free-radical scavenging activities [101, 102] (Fig. 7). Similarly, previous studies have found that the brain, liver, and lung tissues of Brandt’s voles exhibit a certain degree of antioxidant defense ability when exposed to chronic hypoxia, which may be related to their intermittent hypoxic life history [103, 104].

Polyunsaturated fatty acids play a crucial role in inflammatory responses, immune function, oxidative stress, and blood pressure regulation [105]. In the skeletal muscle of *M. musculus* under hypoxia, several polyunsaturated fatty acids, such as alpha-linolenic acid, 12 (S)-HETE, 15-HETE, and 12, 13-EODE, were downregulated, which may not be conducive to angiogenesis. Among these, alpha-linolenic acid has anti-inflammatory properties, improves blood pressure, and supports vascular health [106]. In addition, 12 (S)-HETE has been found to promote endothelial cell migration and angiogenesis while inhibiting cell apoptosis under hypoxic conditions [107]. Furthermore, 15-HETA stimulates vascular endothelial growth factor (VEGF) production through various cell signaling pathways, promoting endothelial cell proliferation, migration, and angiogenesis [108]. The downregulation of these metabolites was consistent with the inhibition of arachidonic acid metabolism indicated by our GSEA results (Fig. 4F). In addition, we observed indoxylsulfuric acid accumulation in the skeletal muscle of *M. musculus*. As a myotoxin, indoxylsulfuric acid induces ROS production in skeletal muscle, triggering an increase in inflammatory cytokines, such as tumor necrosis factor-α, and upregulating genes associated with muscle atrophy [109]. Furthermore, indoxylsulfuric acid reduces glutathione levels, thereby rendering cells susceptible to oxidative stress [110], which corresponds to the downregulation of *Oxr1*, encoding antioxidant protein 1, in transcriptome analysis results [23]. However, we also identified several DAMs that may assist *M. musculus* in coping with hypoxia. The upregulation of 3-hydroxyisovaleric acid, a natural leucine metabolite, has been shown to reduce protein degradation and prevent muscle atrophy [111]. Succinic anhydride, which was upregulated in our analysis, exhibits significant antihypoxic activity and can reduce lactic acid accumulation during hypoxia [112]. In addition, kynurenic acid, the final product of tryptophan metabolism that was upregulated in this context, scavenges ROS and reduces cell oxidative damage [52] (Fig. 7).

## Supplementary Information

Supplementary Material1.

Supplementary Material2.

Supplementary Material3.

Supplementary Material4.

Supplementary Material5.

Supplementary Material6.

Supplementary Material7.

Supplementary Material8.

Supplementary Material9.

Supplementary Material10.

Supplementary Material11.

Supplementary Material12.

Supplementary Material13.

## Data Availability

RNA-Seq data are available in the NCBI database (Bioproject ID: PRJNA993829).
