# Supplementary figures and images for: Variation in metabolic pattern regulation under hypoxic conditions: a comparative study of rodents distributed at different altitudes
Source: Front Zool. 2025 Oct 1;22:27. doi: 10.1186/s12983-025-00582-2 (PMC12487112; doi:10.1186/s12983-025-00582-2)

**Fig. S1**


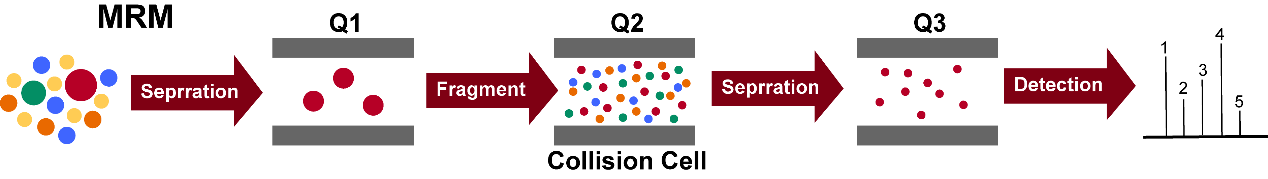


**Fig. S2**


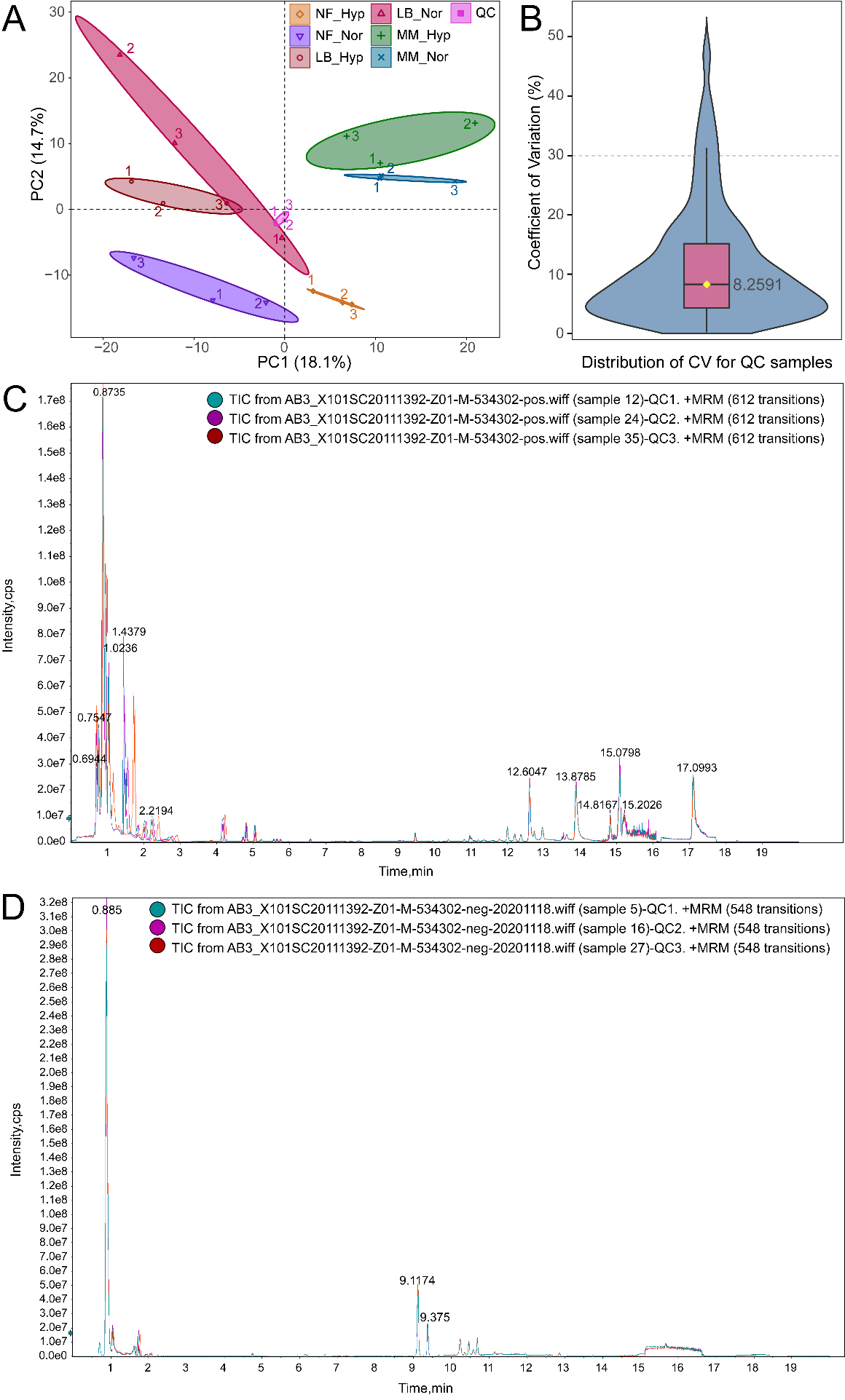


**Fig. S3**


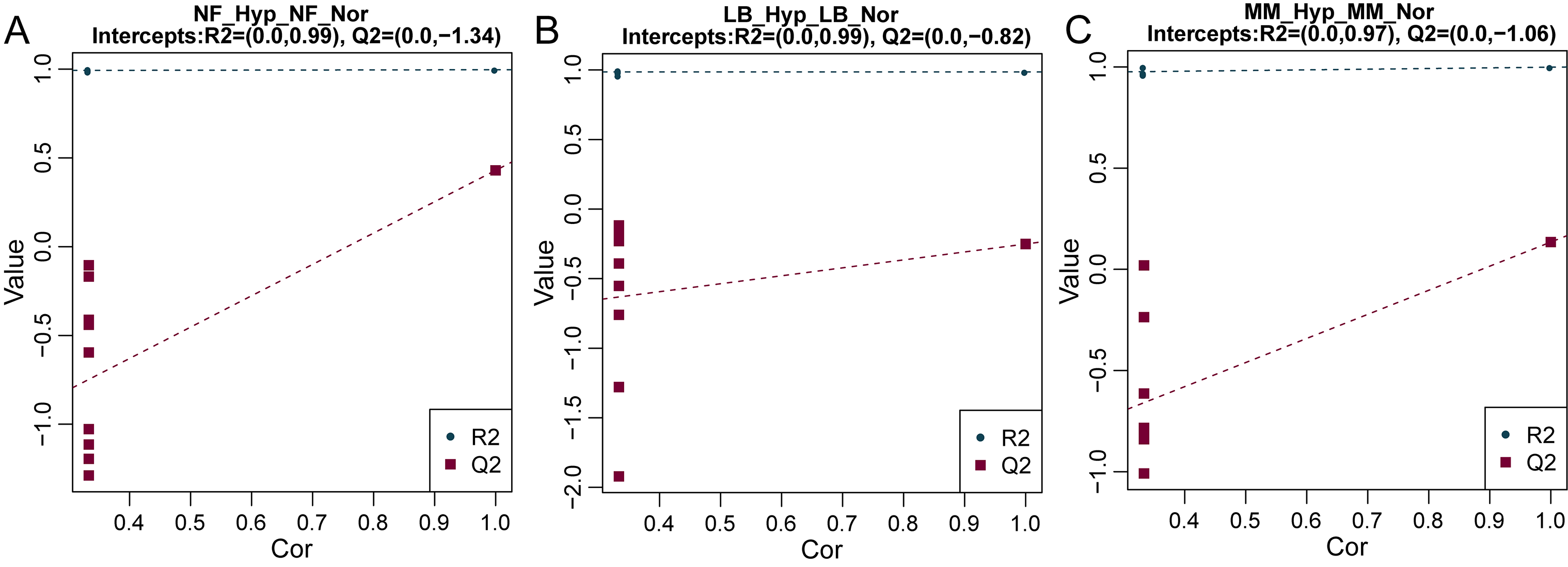


**Fig. S4**


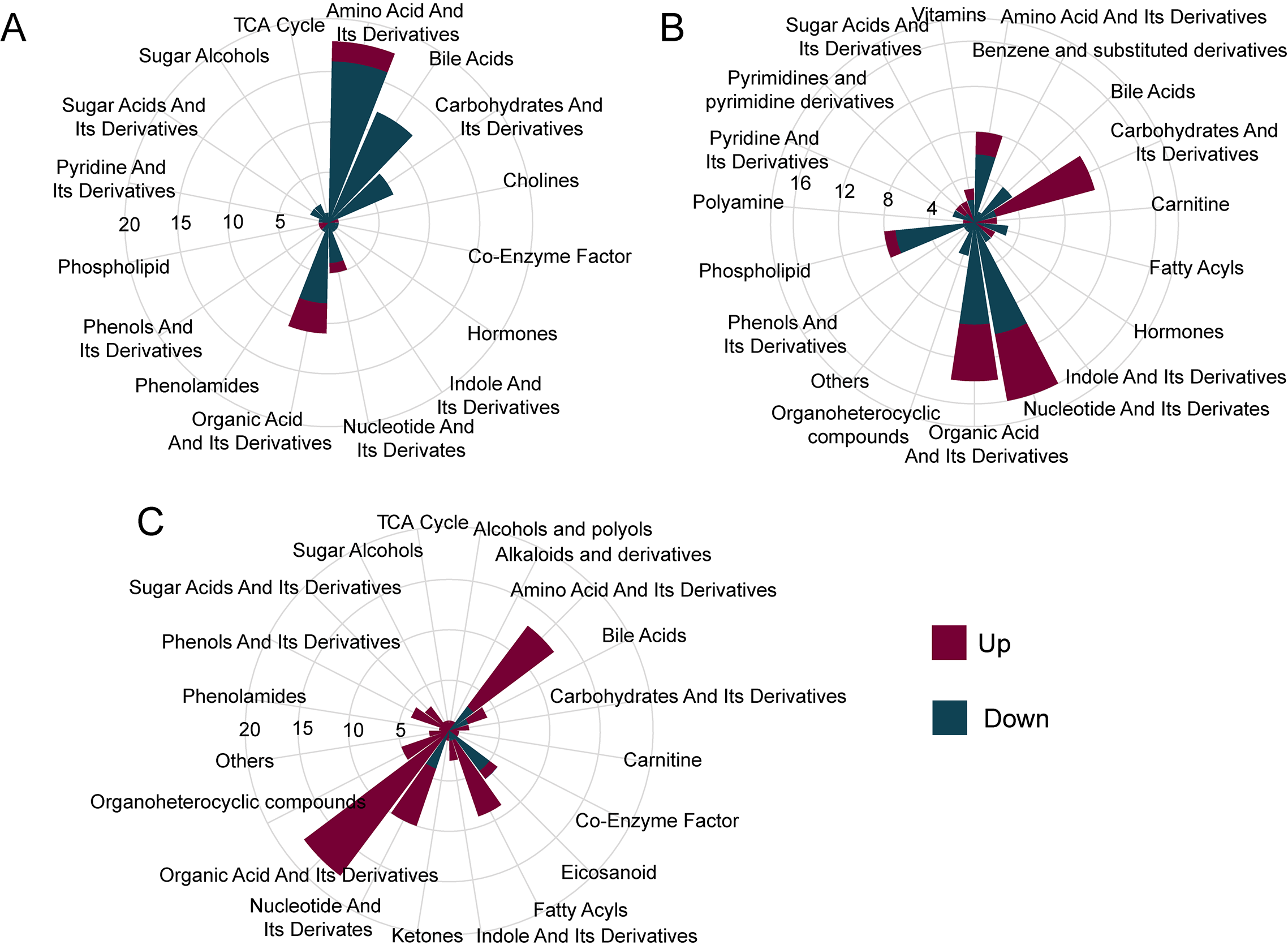

Supplement: Supplementary file 1 — Supplementary Material1. [file 12983_2025_582_MOESM1_ESM.docx]
